# Supplementary material for: Oxone®-Mediated TEMPO-Oxidized Cellulose Nanomaterial Ultrafiltration and Dialysis Mixed-Matrix Hollow Fiber Membranes
Source: Polymers (Basel). 2020 Jun 15;12(6):1348. doi: 10.3390/polym12061348 (PMC7361684; doi:10.3390/polym12061348)
Supplement: Supplementary file 1 [file polymers-12-01348-s001.pdf]

The composition of the three different membranes formulated with the solution made from the sonication protocol (1 wt.% OTO-CNMs, 9 wt.% CTA, and 90 wt.% NMP) for casting and further characterization can be identified in Supplemental Supplemental Table **S** below.

Supplemental Table S1: The compositions of OTO-CNM/CTA membranes based on wt.% for Form I to Form II

Membrane Sample	OTO-CNM Form I (wt.%)	OTO-CNM Form II (wt.%)	CTA (wt.%)	NMP (wt.%)
(a) Form I	1.0	0.0	9.0	90.0
(b) 50/50	0.5	0.5	9.0	90.0
(c) Form II	0.0	1.0	9.0	90.0
(d) CTA	0.0	0.0	10.0	90.0

Once the final combination of membrane materials was completed, the mixture was sealed in the reagent bottle to avoid the evaporation of the solvent. The bottle was continuously mixed in a bottle roller over 72–120 h at room temperature before casting.

Sieving coefficients plotted logarithmically in Figure 3 can be seen in the Supplemental Table 2 below.

Supplemental Table S2: Membrane Sieving Coefficient Information

Sample	Molecular Weight (Dalton)	Control	Form I	Form II	50/50
BSA	66000	0.13 ± 0.07	0.19 ± 0.11	0.19 ± 0.10	0.31 ± 0.01
Lysozyme	14000	0.88 ± 0.06	0.75 ± 0.03	0.68 ± 0.05	0.77 ± 0.01
Urea	60	1.03 ± 0.03	1.06 ± 0.05	0.99 ± 0.04	1.03 ± 0.03
